# CCL19 reduces tumour burden in a model of advanced lung cancer

**DOI:** 10.1038/sj.bjc.6603061

**Published:** 2006-04-04

**Authors:** S Hillinger, S-C Yang, R K Batra, R M Strieter, W Weder, S M Dubinett, S Sharma

**Affiliations:** 1 Thoracic Surgery, University Hospital Zürich, Zürich, Switzerland; 2 Department of Medicine, David Geffen School of Medicine, University of California Los Angeles, Los Angeles, CA, USA

**Keywords:** lung cancer, immunotherapy, chemokines

## Abstract

Epstein–Barr virus-induced molecule 1 ligand chemokine (CCL19) is a CC chemokine that chemoattracts both dendritic cells (DC) and T lymphocytes. We evaluated the antitumour efficacy of CCL19 in a murine model of spontaneous bronchoalveolar cell carcinoma. These transgenic mice (CC-10 TAg) express the SV40 large T antigen under the Clara Cell promoter, develop bilateral, multifocal, pulmonary carcinomas and die at 4 months owing to progressive pulmonary tumour burden. To mimic therapy in late-stage disease, 3-month-old transgenic mice were treated with recombinant CCL19 (0.5 *μ*g dose^−1^) by intranodal (axillary lymph node region) injection three times per week for 4 weeks. CCL19 treatment led to a marked reduction in tumour burden with extensive mononuclear infiltration of the tumours compared to diluent treated controls. Flow cytometric analyses showed significant increases in CD4 and CD8T cell subsets as well as DC in the lungs of CCL19-treated mice. Lung tissue cytokine profiles showed a shift towards immune stimulatory molecules with a decrease in the immunosuppressive cytokine TGF-*β*. Our findings show that CCL19 may serve as a potential immune stimulator and provide a strong rationale for the evaluation of CCL19 in cancer immunotherapy.

Lung cancer is the leading cause of cancer death in the United States and in the world (Jemal *et al*, 2004). With the existing therapeutic efforts, patients with lung cancer still have a poor prognosis and less than 15% live 5 years. This dismal statistic has changed minimally in the last 20 years and, therefore, new therapeutic strategies are clearly needed.

Effective antitumour responses require both antigen-presenting cells (APC) and lymphocyte effectors (Huang *et al*, 1994). Although lung cancers express tumour antigens (Yu *et al*, 1997), they are ineffective as APCs (Qin *et al*, 1997) because tumour cells often have limited expression of MHC Ags and lack co-stimulatory molecules (Restifo *et al*, 1993). In addition, tumour cells produce immune inhibitory factors that promote escape from immune surveillance (Huang *et al*, 1998; Sharma *et al*, 1999). Consequently, effective anticancer immunity may be achieved by recruiting professional host APC for tumour Ag presentation to promote specific T-cell activation (Miller *et al*, 2000).

Chemokines are a group of homologous, yet functionally divergent proteins that directly mediate leucocyte CXCL9/MIGration and activation and play a role in regulating angiogenesis (Baggiolini *et al*, 1997). Epstein–Barr virus-induced molecule 1 ligand chemokine (ELC/CCL19) is produced by a subset of dendritic cells (DC), and possibly by other non-lymphoid cells, in T-cell areas of lymphoid tissue (Ngo *et al*, 1998). CCL19 ability to chemoattract T cells (Kim *et al*, 1998a, 1998b, 1999; Ngo *et al*, 1998), B cells (Kim *et al*, 1998a, 1999), DC (Dieu *et al*, 1998), macrophage progenitor cells (Kim *et al*, 1998c) and NK cells (Kim *et al*, 1999) is mediated through the specific G protein-coupled seven transmembrane domain chemokine receptor CCR7. Based on the capacity of CCL19 to facilitate co-localisation of both DC and T cells, we and others are evaluating the capacity of CCL19 to reverse tumour-mediated immune suppression and orchestrate effective cell-mediated immune responses. In recent studies, murine breast tumour cells transduced by a retroviral vector expressing CCL19 were rejected *in vivo* by a mechanism that involves both CD4+ and NK cells (Braun *et al*, 2000). Utilising subcutaneous murine lung cancer models, we have previously shown that CCL19 promotes IFN-*γ*-dependent antitumour responses (Hillinger *et al*, 2003).

In this study, utilising a transgenic murine model of spontaneous lung cancer that more closely resembles human lung cancer, we demonstrate that CCL19 mediates potent antitumour responses *in vivo* leading to significant tumour reduction.

## MATERIALS AND METHODS

### CC-10 TAg mice

The transgenic CC-10 TAg mice, in which the SV40 large T antigen is expressed under control of the murine Clara cell-specific promoter, were used in these studies (Magdaleno *et al*, 1997). Mice expressing the transgene developed diffuse bilateral bronchoalveolar carcinoma in the lung. Tumours were evident bilaterally by microscopic examination as early as 4 weeks of age. After 3 months of age, the bronchoalveolar pattern of tumour growth coalesced to form multiple bilateral tumour nodules. The CC-10 TAg transgenic mice had an average lifespan of 4 months. Extra-thoracic metastases were not noted. Breeding pairs for these mice were generously provided by Francesco J DeMayo (Baylor College of Medicine, Houston, TX, USA). Transgenic mice were bred at the West Los Angeles Veteran Affairs vivarium and maintained in the West Los Angeles Veterans Administration Association for Assessment and Accreditation of Laboratory Animal Care-accredited Animal Research Facility. Before each experiment utilising the CC-10 TAg transgenic mice, presence of the transgene was confirmed by PCR of mouse-tail biopsies as described previously (Sharma *et al*, 2001). All of the experiments used pathogen-free CC-10 TAg transgenic mice at 3 months of age. All procedures were carried out in accordance with the Guidelines for the Welfare of Animals in Experimental Neoplasia (UKCCCR, 1998).

### The CCL19 therapeutic model in CC-10 TAg mice

CC-10 TAg transgenic mice were injected in the axillary lymph node region with murine recombinant CCL19 (0.5 *μ*g injection^−1^; Pepro Tech, Rocky Hill, NJ, USA) or normal saline diluent, which contained equivalent amounts of murine serum albumin (Sigma Chemical Co., St Louis, MO, USA) as an irrelevant protein for control injections. Starting at 12 weeks of age, CCL19 or control injections were administered three times per week for 4 weeks. At the age of 4 months, mice were killed, and lungs were isolated for quantification of tumour surface area. Tumour burden was assessed by microscopic examination of H&E-stained sections with a calibrated graticule (a 1-cm^2^ grid subdivided into 100 1-mm^2^ squares). A grid square with tumour occupying >50% of its area was scored as positive, and the total number of positive squares was determined as described previously (Sharma *et al*, 1999). Ten separate fields from four histological sections of the lungs were examined under high power (× 20 objective).

### Cytokine determination from tumour nodules and spleens by ELISA

The cytokine profiles in lung tumours and spleens were determined in both CCL19 and diluent-treated mice as described previously (Sharma *et al*, 1999). The lungs were harvested and cut into small pieces and homogenised for cytokine determinations (Bellco, Vineland, NJ, USA). Spleens from the various treatment groups were isolated, teased apart, RBC depleted with ddH_2_O, and brought to tonicity with 1 × PBS. Spleen cells were cultured for 24 h and cytokines and PGE-2 determined in the culture supernatants. The amounts of IL-10, IL-12, GM-CSF, IFN-*γ*, TGF-*β*, CXCL9/MIG, and CXCL10/IP-10 were quantified by ELISA and that of PGE-2 by EIA. Tumour-derived cytokine and PGE-2 concentrations were corrected for total protein by Bradford assay (Sigma Chemical Co., St Louis, MO, USA) and the results expressed as pg mg^−1^ of total protein. For the TGF-*β* ELISA measurements, samples were acidified and hence the active form of TGF-*β* was measured. The sensitivities of the IL-10, GM-CSF, IFN-*γ*, TGF-*β*, IL-12, and CXCL10 ELISA were 15 pg ml^−1^. The plates were read at 490 nm with a Molecular Devices Microplate reader (Sunnyvale, CA, USA).

### PGE2 EIA

The concentrations of PGE2 were determined using a kit from Cayman Chemical Co. (Ann Arbor, MI, USA) according to the manufacturer's instructions as described previously (Huang *et al*, 1998). The EIA plates were read by a Molecular Devices Microplate reader (Sunnyvale, CA, USA).

### Flow cytometry

For flow cytometric experiments, two or three fluorochromes (PE, FITC, and Tri-color; PharMingen) were used to gate on the CD3 T lymphocytes in the percol purified leucocyte populations from tumour nodules as described previously (Yang *et al*, 2004). Dendritic cells were defined as the double-stained CD11c+DEC205+ bright populations within the purified leucocyte populations from the tumour nodules. T-regulatory cells in the purified leucocyte populations from the tumour nodules were stained for the cell surface markers CD4 and CD25. Flow cytometric analyses were performed on a FACScan flow cytometer (Becton Dickinson, San Jose, CA, USA) in the University of California, Los Angeles, Jonsson Cancer Center Flow Cytometry Core Facility. Between 10 000 and 15 000 gated events were collected and analysed using Cell Quest software (Becton Dickinson).

## RESULTS

**Figure 1:**
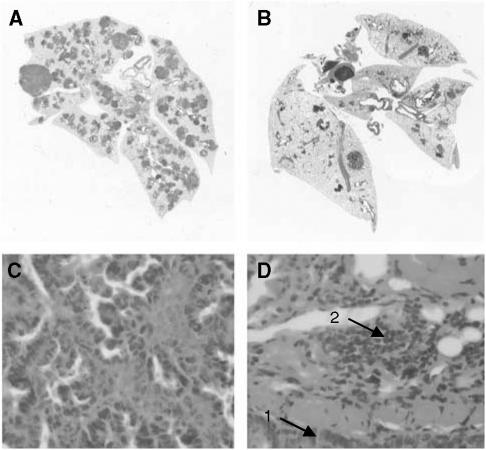
CCL19 mediates potent antitumour responses in a murine model of spontaneous lung cancer. Three-month-old CC-10 TAg mice were injected with CCL19 (0.5*μ*g injection^−1^) or the same concentration of murine serum albumin in the axillary lymph node region three times a week for 4 weeks. H&E staining of paraffin-embedded lung tumour sections from control-treated mice evidenced large tumour masses throughout both lungs with few mononuclear infiltrations (**A** and **C**). In contrast, CCL19-treated mice evidenced a marked reduction in tumour burden (**B** and **D**) with prominent mononuclear infiltrations (1, tumour; 2, mononuclear cells). Tumour burden was quantified within the lung by microscopy of H&E-stained paraffin-embedded sections.

**Figure 2:**
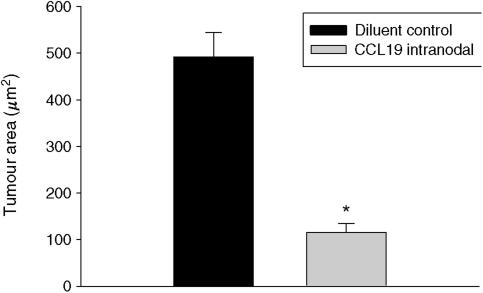
CCL19 mediates potent antitumour responses in a murine model of spontaneous lung cancer. There was reduced tumour burden in CCL19-treated CC-10 mice compared with the diluent-treated control group . ^*^P<0.001 compared with control tumour group, *n*=8 mice/group.

**Figure 3:**
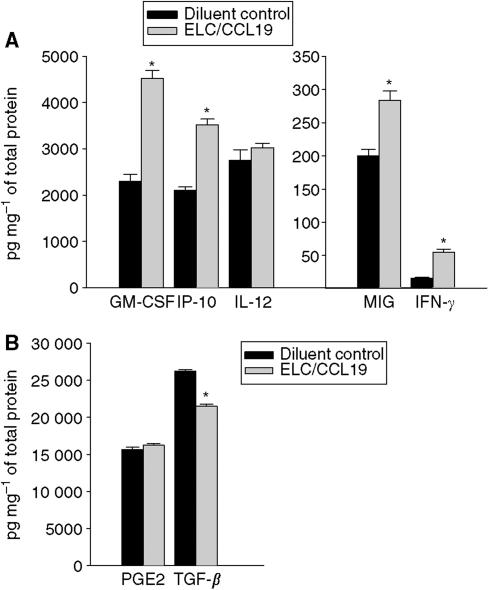
CCL19 therapy leads to an induction of Th1 cytokines and antiangiogenic chemokines and a decrease in TGF-*β*. Following axillary lymph node region injection of CCL19, pulmonary cytokine profiles in CC-10 TAg mice were determined and compared with those in diluent-treated tumour-bearing control mice. Compared with lungs from diluent-treated CC-10 tumour-bearing mice, CC-10 TAg mice treated with CCL19 had significant increase in GM-CSF, IFN-*γ*, CXCL9/MIG, and CXCL10/IP-10 but a decrease in TGF-*β* (**A** and **B**). ^*^P<0.01 compared with diluent-treated CC-10 TAg mice, *n*=6 mice per group.

**Figure 4:**
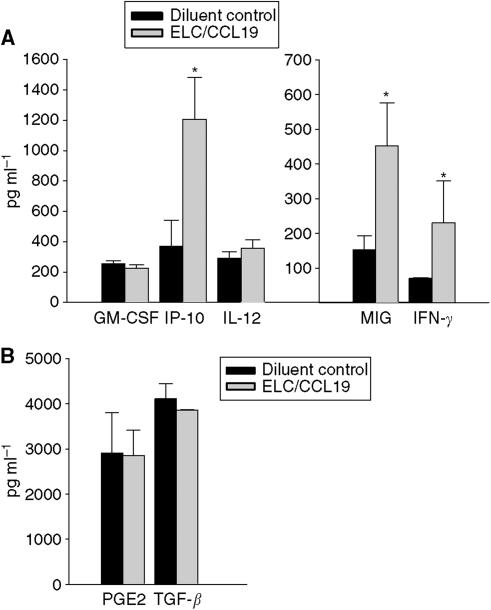
CCL19 therapy leads to an induction of Th1 cytokines and antiangiogenic chemokines and a decrease in TGF-*β*. Following axillary lymph node region injection of CCL19, spleen cytokine profiles in CC-10 TAg mice were determined and compared with those in diluent-treated tumour-bearing control mice. Compared with diluent-treated CC-10 TAg mice, splenocytes from CCL19-treated CC-10 TAg mice had significant increases in IFN-*γ*, CXCL9/MIG, and CXCL10/IP-10. ^*^P<0.01 compared with diluent-treated CC-10 TAg mice, *n*=6 mice per group.

CCL19 has potent systemic antitumour responses *in vivo* (Figures 1 and 2); it enhances the frequency of T-cell subsets and DCs at the tumour sites (Table 1); it promotes type 1 cytokine and antiangiogenic chemokine release as well as a decline in the immunosuppressive cytokine TGF-*β* (Figures 3 and 4).

### CCL19 mediates potent antitumour responses in a murine model of spontaneous bronchoalveolar carcinoma

We evaluated the antitumour efficacy of CCL19 in a late-stage spontaneous bronchoalveolar cell carcinoma model. CCL19 (0.5 *μ*g injection^−1^) or the same concentration of murine serum albumin was injected in the axillary lymph node region beginning at 12 weeks of age, three times per week and continuing for 4 weeks. At 4 months when the control mice started to succumb because of progressive lung tumour growth, mice were killed in all of the treatment groups, and lungs were isolated and paraffin embedded. H&E staining of paraffin-embedded lung tumour sections from control-treated mice revealed large tumour masses throughout both lungs with minimal lymphocytic infiltration (Figure 1A and C). In contrast, CCL19-treated mice had significantly smaller tumour nodules with extensive lymphocytic infiltration (Figure 1B and D). Mice treated with CCL19 had a marked reduction in pulmonary tumour burden (130±15 *μ*m^2^) as compared with diluent-treated control mice (495±40 *μ*m^2^) (Figure 2) (*P*<0.001).

### CCL19 treatment of CC-10 TAg mice leads to enhanced DC and T-cell infiltrates at tumour sites

To determine if the reduced tumour burden was accompanied by increases in T-cell and DC-infiltrates following CCL19 therapy, flow cytometric analyses was performed on percol purified leucocyte populations of tumour nodules. Compared with the diluent-treated control group, the CCL19-treated CC-10 TAg mice showed significant increases in the frequency of CD4 (20%), CD8 (50%) and CD11c+DEC205+ DC (70%) and a decrease in CD4+CD25+ Treg (20%) cells at the tumour site (Table 1). ^*^P<0.001 as compared to diluent-treated control.

### CCL19 therapy promotes type 1 cytokines and antiangiogenic chemokine release but a decline in the immunosuppressive cytokine TGF-*β*

Tumour progression can be modified by host cytokine profiles; hence, we measured the cytokine production from tumour sites and spleens following therapy. Lungs and spleens were evaluated for the presence of IFN-*γ*, GM-CSF, IL-12, CXCL9, CXCL10, and TGF-*β* by ELISA, and PGE2 by EIA. Compared to diluent-treated controls, the treatment group receiving CCL19 had a significant increase in type 1 cytokines (GM-CSF, IFN-*γ*) and antiangiogenic chemokines (CXCL9, CXCL10) and a decrease in the immunosuppressive cytokine TGF-*β* at the tumour sites. Compared with lungs from the diluent-treated group, CC-10 TAg mice treated with CCL19 had a significant reduction in TGF-*β* (1.3-fold, *P*<0.05). This was coupled with an increase in GM-CSF (two-fold, *P*<0.01), IFN-*γ* (4.2-fold, *P*<0.01), CXCL9 (1.4-fold, *P*<0.01), and CXCL10 (1.7-fold, *P*<0.05) within the tumour microenvironment (Figure 3A and B). Moreover, a systemic effect was evident as similar cytokine patterns were also observed in the spleens of CCL19-treated mice. Thus, compared with the diluent-treated group, splenocytes from CCL19-treated CC-10 TAg mice revealed an increase in IFN-*γ* (2.8-fold, *P*<0.001), CXCL9 (3.2-fold, *P*<0.05), and CXCL10 (three-fold, *P*<0.05) (Figure 4A and B).

## DISCUSSION

Host APC are critical for the cross-presentation of tumour antigens (Huang *et al*, 1994). However, tumours have the capacity to limit APC maturation, function, and infiltration of the tumour site (Gabrilovich *et al*, 1996, 1997, 1998; Qin *et al*, 1997). Thus, molecules that attract host APC and T cells could serve as potent agents for cancer immunotherapy.

CCL19 is produced by a subset of DCs, and possibly by other non-lymphoid cells; in T-cell areas of lymphoid tissue (Ngo *et al*, 1998), it strongly attracts naïve T cells and DCs. Because DCs are potent APCs that function as principal activators of T cells, CCL19's capacity to facilitate the colocalisation of both DC and T cells may reverse tumour-mediated immune suppression and orchestrate effective cell-mediated immune responses. In addition, CCL19 is a potent inducer of T-cell proliferation and programmes DCs for the induction of T helper (Th) 1 rather than Th2 responses (Marsland *et al*, 2005). Based on these properties, we speculated that CCL19 would be an important protein for evaluation in cancer immunotherapy.

In most models reported previously, the antitumour efficacy of chemokines was determined using transplantable murine or human tumours propagated at s.c. sites. We embarked on the current studies to determine the antitumour properties of CCL19 in a clinically relevant model of lung cancer in which bronchoalveolar carcinomas develop in an organ-specific manner. The antitumour activity of CCL19 was determined in the spontaneous model for lung cancer by injecting recombinant CCL19 into the axillary lymph node region of the transgenic mice. In many clinical situations, access to lymph node sites for injection may also be more readily achievable than intratumoral administration. The efficacy of injecting immune stimulators in the vicinity of the lymph nodes for the treatment of cancer has been demonstrated previously; vaccination with tumour cell–DC hybrids in the lymph node region led to regression of human metastatic renal cell carcinoma (Kugler *et al*, 2000). Our rationale for injecting CCL19 into the lymph node region was to colocalise DC and T cells to the lymph nodes where they can prime specific antitumour immune responses, and our results show that this approach works. CCL19 injected into the axillary lymph node region evidenced potent antitumour responses, with reduced tumour burden as compared to mice receiving diluent control injections. The reduced tumour burden in CCL19-treated mice was accompanied by extensive lymphocyte as well as DC infiltrates of the tumour sites (Figure 1 and Table 1). Although the tumours in the transgenic mice express the SV40 T antigen, we do not think that the CCL19-mediated increase in T and DC infiltrates into the tumour sites is in response to the viral antigen because in order for the tumours to grow in this model, the T cells must be tolerised to the SV 40 T antigen. In a recent study (Yang *et al*, 2006), utilising the CCR7 ligand CCL21, we have shown that cytolytic T-cell responses were enhanced to autologous CC-10 tumours but not to syngeneic control MLE-12 tumours that also express the SV40 T antigen. This suggests that the T-cell reactivity against tumours in this model in response to CCR7 ligand therapy is not owing to the viral SV40 T antigen but to tumour-specific antigens.

It is well documented that successful immunotherapy shifts tumour-specific T-cell responses from a type 2 to a type 1 cytokine profile (Hu *et al*, 1998). T helper 1 responses depend on IFN-*γ* to mediate a range of biological effects that facilitate anticancer immunity. CXCL9/MIG/CXCL9 and CXCL10/IP-10/CXCL10 are antiangiogenic CXC chemokines induced by IFN-*γ* that chemoattract activated T cells expressing the CXCR3 chemokine receptor (Loetscher *et al*, 1996) and are known to have potent antitumour and properties (Brunda *et al*, 1993; Luster and Leder, 1993; Arenberg *et al*, 1996; Sgadari *et al*, 1997). The tumour sites of CCL19-treated mice revealed significant increases in IFN-*γ*, CXCL10/CXCL10/IP-10, CXCL9/CXCL9/MIG, and GM-CSF. The induction of CXCL9/CXCL9/MIG and CXCL10/CXCL10/IP-10 may be responsible in part for the tumour reduction following CCL19 administration. Hence, the tumour reductions observed in this model may be owing to T-cell-dependent immune resposes as well as participation by T cells secreting IFN-*γ* in inhibiting angiogenesis (Tannenbaum *et al*, 1998). Hence, an increase in IFN-*γ* at the tumour site of CCL19-treated mice could explain the relative increases in CXCL10/IP-10 and CXCL9/MIG. Both CXCL9/MIG and CXCL10/IP-10 are chemotactic for stimulated CXCR3-expressing T lymphocytes that could further amplify IFN-*γ* at the tumour site (Farber, 1997). An increase in GM-CSF in CCL19-treated mice could enhance DC maturation and antigen presentation (Banchereau and Steinman, 1998). Further studies are necessary to precisely define the host cytokines that are critical to the CCL19-mediated antitumour response. In addition, CCL19-treated tumour-bearing mice showed significant reductions in TGF-*β* at the tumour sites; TGF-*β* is an immune inhibitory cytokine that may potently suppress Ag and presentation, antagonise CTL generation and macrophage activation (Bellone *et al*, 1999). Thus, possible benefits of a CCL19-mediated decrease in TGF-*β* include promotion of antigen presentation and CTL generation (Bellone *et al*, 1999) as well as a limitation of angiogenesis (Fajardo *et al*, 1996; Tsujii *et al*, 1998). The increase in the type 1 cytokines was not limited to the lung but was evident systemically. CCL19 treatment of CC-10 TAg transgenic mice led to systemic increases in type I cytokines and antiangiogenic chemokines. Hence, splenocytes from CCL19-treated CC-10 TAg mice had an increase in GM-CSF, CXCL9/MIG, and CXCL10/IP-10 as compared with diluent-treated CC-10 TAg mice.

Regulatory T cells have been documented to function as suppressor cells, and may play a role in the progression of cancer (Antony and Restifo, 2005). Failure of tumour immunosurveillance or enhanced tumour growth could be owing to an increase of regulatory T cells producing inhibitory cytokines or directly inhibiting immunity via specific cellular interactions at the tumour site (Antony and Restifo, 2002; Shevach, 2002). There was a modest decrease in the frequency of CD4^+^CD25^+^ Treg population at the tumour site following CCL19 therapy. The decrease in Treg cells may not be physiologically relevant in terms of numbers but may be in terms of function. Further studies are needed to define the functional significance of the decrease in the Treg population at the tumour site.

Taken together, the current study indicates that CCL19 injected in the axillary lymph node region in the late-stage spontaneous lung cancer model leads to the generation of effective antitumour responses. The potent antitumour properties demonstrated in this model of spontaneous bronchoalveolar carcinoma provide a strong rationale for additional evaluation of CCL19 regulation of tumour immunity and its use in immunotherapy for lung cancer.

## Figures and Tables

**Table 1:** CCL19 enhances influx of T-cell subsets and DC at the tumour sites

**Phenotype**	**Control**	**ELC/CCL19**
CD4+	17±0.7	21±0.9^*^
CD8+	38±2.1	56±1.8^*^
CD4+CD25+	11±0.2	9±0.1^*^
CD11c+DEC205+	9±1.0	15±1.2^*^

DC=dendritic cells.

Percol purified leucocytes from the digested tumour nodules from CCL19 and diluent-treated CC-10 TAg mice were identified as lymphocytes or DC by staining with cell surface markers. Cell surface staining or T-cell markers CD4, CD8, CD4+CD25+ as well as the DC markers CD11c and DEC205 were evaluated by flow cytometry; 10 000–15 000 gated events were collected and analysed using Cell Quest software. CCL19 treatment led to an increase in the frequency of CD4^+^, CD8^+^, CD11c+DEC205+ DC and a modest decrease in CD4+CD25+ Treg cells compared to the diluent-treated control (^*^P<0.001; *n*=8 mice per group.) These experiments were repeated twice. Unit: percentage of leucocytes±s.e.
